# Primary Signet Ring Cell Carcinoma of the Prostate: A Rare Case Report

**DOI:** 10.3390/jcm7080218

**Published:** 2018-08-15

**Authors:** Alper Gök, Can Tuygun, Müge Akmansu, Ayşegül Aksakal Uslu, Ibrahim Güven Kartal, Fatih Sandikçi, Osman Raif Karabacak, Azmi Levent Sağnak, Hikmet Topaloğlu, Hamit Ersoy

**Affiliations:** 1 Department of Urology, University of Health Sciences, Dışkapı Yıldırım Beyazıt Training and Research Hospital, Ankara 06030, Turkey; drct36@gmail.com (C.T.); igk84@hotmail.com (I.G.K.); drfatihsandikci61@hotmail.com (F.S.); osmanraifkarabacak@mynet.com (O.R.K.); leventsagnak@gmail.com (A.L.S.); hikmettopaloglu@hotmail.com (H.T.); hamitersoy@gmail.com (H.E.); 2 Department of Radiation Oncology, Gazi University Medical Faculty, Ankara 06560, Turkey; mugeakmansu@gmail.com; 3 Department of Pathology, University of Health Sciences, Dışkapı Yıldırım Beyazıt Training and Research Hospital, Ankara 06030, Turkey; guluslu@gmail.com

**Keywords:** prostate, carcinoma, signet ring cell, radiotherapy

## Abstract

Primary prostatic signet ring cell carcinoma is a rare form of cancer with a poor prognosis, which is generally treated with a traditional prostate adenocarcinoma therapy. This case report presents a 70-year-old diagnosed with primary prostatic signet ring cell carcinoma, treated with a combination of radiotherapy and hormone therapy and a 16 month survival without an evidence of the disease at follow up.

## 1. Introduction

Signet ring cell carcinoma (SRCC) is most frequently observed in the stomach and colon. It occurs less frequently in the pancreas, breast, thyroid and bladder. Primary SRCC of prostate is quite rare. Until recently, approximately 60 cases with primary prostate SRCC have been presented in the literature. The prognosis of primary prostate SRCC is poor with no clear treatment approach due to low number of cases [1]. This report presents a case report of a patient recently treated with a quite rare histological variant of prostate adenocarcinoma.

## 2. Case Report

**Figure 1:**
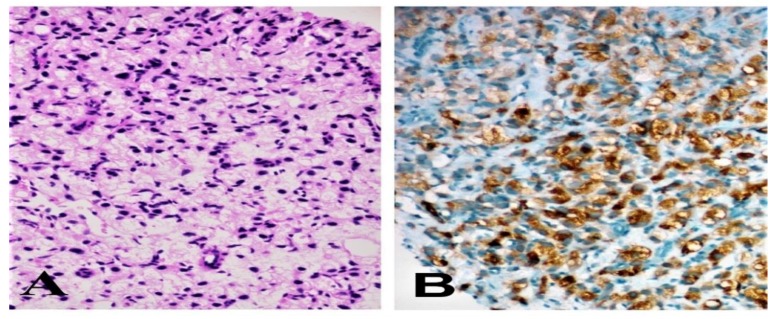
Specimen with hematoxylin and eosin staining of tumor cells (magnification ×200) (**A**); specimen staining positively for PSA (magnification ×200) (**B**).

A 70-year-old patient with lower urinary tract symptoms and a PSA level of 7.26 ng/mL was presented at the hospital. The digital rectal examination did not reveal any hardness or nodules. A 12-core prostate biopsy revealed a Gleason grade 5 + 5 prostate adenocarcinoma in all quadrants and a signet ring cell component in nearly half of all quadrants (Figure 1A). Immunohistochemical analysis was negative for leukocyte common antigen (LCA) and alfa smooth muscle actin (ASMA), but was positive for PSA (Figure 1B) and prostate specific acid phosphatase (PSAP) staining. Cytoplasmic immunostaining with PSA in tumor cells supports the origin of prostatic adenocarcinoma. In this case, there was a strong cytoplasmic staining with PSA in signet-ring-like cells. Colonoscopy and endoscopy were performed in order to exclude metastasis from the gastrointestinal (GI) tract to the prostate due to the signet ring cell component. Biopsies were taken from suspicious areas of GI tract, however no malignity was observed. Pancreas and other abdominal organs were evaluated by a computed tomography (CT) scan, and no pathological signs were found. Furthermore, whole body bone scintigraphy also did not found any metastasis. Based on these findings the patient was diagnosed with non-metastatic disease. A therapy with an LHRH analogue (Goserelin acetate, 10.8 mg, subcutaneous) and image-guided radiotherapy (a daily dose of 200 cGy/fr, 6MV-X-ray beams were used, 7200 cGy to prostate, 6600 cGy to vesicula seminalis, 5000 cGy to bilateral pelvic lymph node region were delivered in 34 fractions) was performed. Serum PSA level which was 7.26 ng/mL before hormonotherapy regressed to 0.37 ng/mL three months after initiation of HT. Serum PSA level which was 0.37 ng/mL before radiotherapy was detected to decrease to 0.32 ng/mL after RT. The patient had a serum PSA level of 0.12 ng/mL and no evidence of the disease was found at 16 months after the start of the therapy.

## 3. Discussion

The signet ring appearance in the cells occurs because the nucleus is pushed to the periphery of the cell by large intracytoplasmic vacuoles. SRCC is most commonly observed in the GI tract. Therefore, when SRCC is detected in the prostate, endoscopy, colonoscopy and abdominal CT scan are needed to exclude metastasis. This case presented here did not showed any GI tract pathological signs. Based on these findings we made the diagnosis of primary prostatic SRCC. Some studies stated that signet ring cells must constitute of at least 20–25% of the tumor to be able to have a diagnosis of primary prostatic SRCC, although other studies stated that a certain ratio of cells was not needed for diagnosis [2,3,4,5]. In this case, the signet ring cell component constituted almost 50% of the tumor. Primary prostatic SRCC is frequently accompanied by high grade prostate adenocarcinoma patterns, therefore it might be a variant of a high-grade adenocarcinoma rather than a separate pathological diagnosis [1,6]. It should not be ignored that an appearance similar to signet rings could be formed in smooth muscle cells and lymphocytes of the prostate after needle biopsies and transurethral resections. In order to rule out such a situation, it needs to be demonstrated that the sample was not stained with LCA and ASMA in immunohistochemical analysis [2]. Immunohistochemistry shows that primary prostatic SRCC cases are 87% positive for PSA/PSAP staining while this cancer is less frequently positive for Periodic acid-schiff (50%), Alcian blue (44%), mucicarmine (40%) and Carcinoembryonic antigen (20%) staining [1]. In this case, immunohistochemistry revealed positive staining for PSA and PSAP whereas LCA and ASMA staining was negative. In the present case, strongly staining with PSA in signet-ring-like cells also suggests that the primary origin of the tumor is prostate tissue.

In the literature, the median age for prostatic SRCC is around 68 years, which is comparable to the currently reported 70 years [1,2]. At the time of diagnosis, most patients have locally advanced or metastatic disease leading to a poor disease prognosis [1,2]. The study by Fujita et al., showed that only the disease stage at the time of diagnosis was associated with the survival, not serum PSA levels nor applied therapy modalities [2]. Furthermore, they showed that the survival rates after the initial diagnosis was 82.3% in the first year, 54.7% in the third year and 11.7% in the fifth year [2]. Saito et al. showed that only 27.3% of patients with prostate SRCC had a 3-year survival rate, with no survival at 5 years [7]. Warner et al. showed an average survival time of 29 months [1].

Due to the rare frequency of prostate SRCC, no standard treatment management is available. The literature shows that most primary prostatic SRCCs are treated with hormone therapy (HT), radiotherapy (RT) and radical prostatectomy (RP) or combinations of these therapies [1,2,3,4,5,6,7]. One study showed a successful result with GI cancer chemotherapy (FOLFOX) [8]. Warner et al. showed the best survival with HT+RP and HT + RT combinations treatments [1]. Yoshimura et al. reported that patients with primary prostatic SRCC survived 100 months after the start of HT+RT combination therapy without any evidence of the disease in their control follow-up [9]. Lilleby et al. reported that they achieved local control and remission in one of their patients 12 months after the start of HT+RT combination therapy [10]. A study in the Mayo Clinic with 27,983 patients diagnosed with prostate cancer showed only nine cases with SRCC [1]. One of these patients was treated with HT+RT combination and had survival without any evidence of disease at a 4th year control visit [1]. The case presented here also achieved a survival of 16 months after the start of HT+RT combination therapy without any evidence of the disease at follow-up.

## 4. Conclusions

There is no standardized therapy for prostate SRCC due to the fact that it is a rare disease. However, application of an aggressive multi-modal therapy seems reasonable because of the poor prognosis of the disease. Radiotherapy combination applied together with hormonal therapy may be an appropriate alternative therapy for prostate SRCC treatment.
